# Deletion of NF-kB p50 alters murine glioblastoma tumor-associated macrophage polarization, reduces tumor growth and prolongs survival

**DOI:** 10.1186/2051-1426-3-S2-P395

**Published:** 2015-11-04

**Authors:** Theresa Barberi, Allison Martin, Sarah Harris-Bookman, Michael Lim, Alan D Friedman

**Affiliations:** 1 Johns Hopkins University School of Medicine, Baltimore, MD, USA

## 

Glioblastoma multiforme (GBM) is a uniformly fatal brain tumor. The GBM microenvironment includes abundant tumor-associated macrophages (TAMs) that predominantly assume a pro-tumor “M2” phenotype rather than a pro-inflammatory “M1” phenotype. TAMs suppress T cell activation, produce VEGF, secrete proliferative factors, and encourage tumor invasion and metastasis. The inhibitory p50 subunit of the NF-kB transcription factor exhibits markedly increased nuclear expression in TAMs and M2-polarized macrophages [1], and p50 knockdown or deletion suppresses expression of M2-associated factors [1-3]. We hypothesize that absence of TAM p50 will convert TAMs to a pro-inflammatory M1 phenotype that will reduce tumor growth and prolong survival.

The murine glioma cell line GL261-Luc was intracranially implanted into wild-type and p50(-/-) mice. Tumors grew 6-fold slower in p50(-/-) compared with wild-type mice, and p50(-/-) mice exhibited significantly increased survival. RT-qPCR analysis of CD11b+ myeloid cells isolated from the brains of tumor-bearing mice revealed decreased M2 marker expression and increased M1 marker expression in the absence of NF-kB p50. Evaluation of tumor-infiltrating T cells indicated that p50(-/-) mice possess decreased Treg cells, and that more p50(-/-) CD4 T cells induce IFNg expression after PMA/Ionomycin stimulation than wild-type CD4 T cells. When M2-polarized p50(-/-) bone marrow-derived macrophages (BMM) are co-cultured *in vitro* with wild-type T cells, they do not suppress T cell proliferation to the same extent as wild-type BMM. These data suggest that NF-kB p50 is an important modulator of the suppressive TAM phenotype in GBM and that deletion of the gene encoding p50 promotes conversion to a pro-inflammatory phenotype that is less tumor-permissive. We anticipate that targeting p50 in combination with immune checkpoint inhibition might prove synergistic in facilitating anti-tumor immunity and tumor regression.

